# Effects of ambient temperature and rumen–protected fat supplementation on growth performance, rumen fermentation and blood parameters during cold season in Korean cattle steers

**DOI:** 10.5713/ajas.18.0621

**Published:** 2018-11-27

**Authors:** Hyeok Joong Kang, Min Yu Piao, Seung Ju Park, Sang Weon Na, Hyun Jin Kim, Myunggi Baik

**Affiliations:** 1 Department of Agricultural Biotechnology and Research Institute of Agriculture and Life Sciences, College of Agriculture and Life Sciences, Seoul National University, Seoul 08826, Korea; 2 Institute of Green Bio Science and Technology, Seoul National University, Pyeongchang 25354, Korea

**Keywords:** Ambient Temperature, Beef Cattle, Blood Metabolites, Cold Stress, Growth, Rumen-protected Fat

## Abstract

**Objective:**

This study was performed to evaluate whether cold ambient temperature and dietary rumen-protected fat (RPF) supplementation affect growth performance, rumen fermentation, and blood parameters in Korean cattle steers.

**Methods:**

Twenty Korean cattle steers (body weight [BW], 550.6±9.14 kg; age, 19.7±0.13 months) were divided into a conventional control diet group (n = 10) and a 0.5% RPF supplementation group (n = 10). Steers were fed a concentrate diet (1.6% BW) and a rice straw diet (1 kg/d) for 16 weeks (January 9 to February 5 [P1], February 6 to March 5 [P2], March 6 to April 3 [P3], and April 4 to May 2 [P4]).

**Results:**

The mean and minimum indoor ambient temperatures in P1 (−3.44°C, −9.40°C) were lower (p<0.001) than those in P3 (5.87°C, −1.86°C) and P4 (11.18°C, 4.28°C). The minimum temperature in P1 fell within the moderate cold-stress (CS) category, as previously reported for dairy cattle, and the minimum temperatures of P2 and P3 were within the mild CS category. Neither month nor RPF supplementation affected the average daily gain or gain-to-feed ratio (p>0.05). Ruminal ammonia nitrogen concentrations were higher (p<0.05) in cold winter than spring. Plasma cortisol concentrations were lower (p<0.05) in the coldest month than in the other months. Serum glucose concentrations were generally higher in colder months than in the other months but were unaffected by RPF supplementation. RPF supplementation increased both total cholesterol (p = 0.004) and high-density lipoprotein (HDL) concentrations (p = 0.03).

**Conclusion:**

Korean cattle may not be significantly affected by moderate CS, considering that the growth performance of cattle remained unchanged, although variations in blood parameters were observed among the studied months. RPF supplementation altered cholesterol and HDL concentrations but did not affect growth performance.

## INTRODUCTION

Exposure of feedlot beef cattle to cold stress reduces growth performance and feed efficiency due to an increase in the maintenance energy required to retain body temperature [1]. Several studies have reported that cold stress negatively influences mortality, the immune system of calves, back fat thickness, the meat quality of beef cattle, and the milk yield of dairy cows [2–4]. Decreased productivity under cold conditions may result from increased basal metabolic intensity [5]. Moreover, cold stress appears to alter the metabolic and digestive status of animals. For example, increases in circulating glucose and non-esterified fatty acids (NEFAs) are likely triggered by elevated metabolic heat production and mobilization of substrates for energy metabolism in adipose tissue and liver [4]. In addition, increases in rumination activity, reticulorumen motility, and the rate of digesta passage have been observed under cold conditions [6]. Such changes in rumen digestive characteristics have been associated with reduced digestion in the reticulorumen, particularly when consuming roughage feed [7]. Rumen fermentation characteristics, including volatile fatty acid (VFA) concentrations, are also altered by cold exposure [8]. However, little information on the effects of cold conditions on growth performance, rumen fermentation, and blood parameters is available for Korean cattle.

Dietary fat supplementation has been reported to alleviate cold stress and increase animal productivity [9]. Due to the relatively higher caloric density of fat, dietary fat supplementation in ruminants may provide additional energy, to meet elevated energy requirements under cold conditions [10]. For example, in lactating dairy cows, dietary fat supplementation was associated with increased circulating glucose, which sufficiently increased substrate availability and thus reduced substrate mobilization from energy reserves [11]. Therefore, dietary fat supplementation could represent an effective strategy to alleviate cold stress. However, little information is available on the effects of fat supplementation on alleviating cold stress in Korean cattle. Dietary rumen-protected fat (RPF) has been used as an energy supplement to enhance the productivity of cattle [12,13]. Therefore, this study was performed to examine the effects of RPF supplementation on the growth performance, rumen fermentation, and blood parameters of Korean cattle steers under cold conditions.

## MATERIALS AND METHODS

### Animals and feeding trial

All experimental procedures involving animals were approved by the Seoul National University Institutional Animal Care and Use Committee (SNUIACUC), Republic of Korea, and conducted in accordance with the Animal Experimental Guidelines of the SNUIACUC. The study was conducted at the University Animal Farm of the College of Agriculture and Life Sciences, Pyeongchang Campus of Seoul National University, South Korea.

In the feedlot trial, 20 Korean cattle steers with an average age of 19.7±0.13 months and body weight (BW) of 550.6±9.14 kg were used. The steers had been fed commercial early fattening stage concentrate using an automatic feeding station (DeLaval Alpro System; DeLaval, Tumba, Sweden) and rice straw, following a conventional feeding program. Water was provided freely. During the 2-week adaptation period before the experiment, all animals were fed an experimental control concentrate (approximately 1.6% BW/animal) and rice straw (1 kg/d/head). Steers were assigned to one of two treatments: the control group and RPF supplementation group. The RPF is prilled form of palm oil, as described [14], and purchased from Ecolex SDN. BHD (Pulau Indah, Selangor, Malyasia). The RPF was composed of 99.63% free fatty acids, including 85.48% palmitic acid (C 16:0), 7.05% oleic acid (C 18:1), 3.45% myristic acid (C 14:0), 1.64% linoleic acid (C 18:2), and 1.04% lauric acid (C 12:0), with an energy density of 9,316 kcal/kg (Haneol Corp., Anseong, Korea). Contents of the distillers dried grains with solubles, corn flour, corn gluten feed, barley stone, and palm oil were also adjusted to make similar non-fiber carbohydrate, neutral detergent fiber (NDF), and acid detergent fiber (ADF) between two diets. Table 1 lists the formula and chemical compositions of the experimental diets. Steers were fed a concentrate diet (1.6% BW) using an automatic feeding station and a rice straw diet (1 kg/d) for 16 weeks (January 9 to February 5 [P1], February 6 to March 5 [P2], March 6 to April 3 [P3], and April 4 to May 2 [P4]). The daily feed intake of the concentrate was automatically recorded online using a computer with the DeLaval Alpro system. Equal amounts of roughage were provided twice daily (08:00 and 18:00) and residual roughage was weighed before the morning feeding. Concentrate and rice straw samples were collected weekly and stored at −20°C until analysis. BW was measured before morning feeding on the start day, at 4-week intervals thereafter.

### Analysis of chemical composition of feed

The chemical compositions (dry matter, crude protein, ether extract, ash, calcium, and phosphorus) of the concentrate and rice straw were determined using the AOAC method [15]. The NDF and ADF contents of the rice straw were analyzed using the sequential method with an ANKOM200 Fiber Analyzer (Ankom Technology Corp., Macedon, NY, USA) and reagents, as described by Van Soest et al [16].

### Blood collection and measurement of ambient temperature

Blood was collected before feeding (after 9 h of fasting) at approximately 09:00 on the start date, and at 4-week intervals thereafter. Blood was collected via jugular venipuncture with both a non-heparinized vacutainer (20 mL; Becton–Dickinson, Franklin Lakes, NJ, USA) and ethylenediaminetetraacetic acid-treated vacutainer (20 mL). Serum and plasma were separated by centrifugation at 1,500×*g* at 4°C for 15 min and stored at −80°C until analysis.

Ambient and climate temperatures and relative humidity inside and outside the barn, were recorded in 1-h intervals using four HOBO data loggers (Onset Computer Corp., Bourne, MA, USA). Minimum, mean, and maximum temperatures and corresponding relative humidity data were chosen at every day, and monthly data were average values of 28 days per month. The experimental farm was covered with a roof and the animals were raised indoors; therefore, the animals were protected from precipitation and direct sunlight. Doors were installed on both sides of the barn, which remained open to allow exposure to cold weather. Thus, low winds may have affected the wind-chill temperature.

### Rumen fluid collection and analysis

After blood collection, rumen fluid was collected before feeding (after 9 h of fasting) using the oral stomach tube method, as described by Shen et al [17]. Rumen fluid pH was measured immediately with a pH meter (Ohaus Corp., Parsippany, NJ, USA). For the VFA analysis, 1 mL of rumen fluid was mixed with 0.2 mL of 25% meta-phosphoric acid and stored at −20°C until analysis, and an additional 30 mL of rumen fluid was stored at −20°C for the ruminal ammonia nitrogen (NH_3_-N) analysis. NH_3_-N concentrations were determined using a modified colorimetric method [18]. VFA concentrations were determined by gas chromatography using an Agilent Tech 7890A (Hewlett Packard, Waldbronn, Germany) with a Supelco fused silica capillary column (30 m×0.25 mm×0.25 μm).

### Blood analysis

The analytical reagents for albumin, glucose, triglycerides (TG), high-density lipoprotein (HDL), low-density lipoprotein (LDL), cholesterol, glutamic oxaloacetic transaminase (GOT), glutamic pyruvate transaminase (GPT), calcium, magnesium, and phosphorus were purchased from JW Medical (Seoul, Korea). The analytical reagents for the NEFA analysis were purchased from Wako Pure Chemical (Osaka, Japan). These parameters were analyzed using an automated chemistry analyzer (7180; Hitachi, Tokyo, Japan). Plasma cortisol was analyzed using a Cortisol Salivary HS Enzyme-linked Immunosorbent Assay Kit (cat. no. SLV4635; DRG Instruments, Marburg, Germany). The intra- and inter-assay coefficients of variation for the cortisol kit were 4.0% and 4.6%, respectively, based on bovine plasma samples. All analytical methods were verified in our laboratory, as reported previously [19].

### Statistical analysis

Differences in climate data by month were analyzed with one-way analysis of variance (ANOVA). Differences in growth performance, blood parameters, and rumen fluid parameters by month and dietary treatment were analyzed using a repeated-measures two-way ANOVA. The statistical model included month, diet, and their interaction. A p value less than 0.05 was considered to indicate significance. All statistical tests were performed using R Studio for Windows software (R Studio, Boston, MA, USA).

## RESULTS AND DISCUSSION

### Climate conditions

The mean (P1: −3.44°C, P2: −0.70°C) and minimum (P1: −9.40°C, P2: −5.5°C) indoor ambient temperatures in January (P1) and February (P2) were lower (p<0.001) than those in April (P4; mean: 11.18°C, minimum: 4.28°C; Table 2). In a previous study, cold stress was categorized as mild (0°C to −6.7°C), moderate (−7.2°C to −13.9°C), or severe (<−13.9°C) under dry-winter-coat conditions in cattle [20]. Therefore, the minimum temperatures of P1 and both P2 and P3 in this study were considered to represent moderate and mild cold-stress (CS) conditions, respectively. In addition, we measured the average temperature during blood collection (08:00 to 10:00). The temperatures were −6.42°C, −0.29°C, 6.79°C, 10.8°C, and 14.0°C on January 9, February 6, March 6, April 4, and May 2, respectively. Then sampling days in January and February were classified as mild CS conditions, while sampling days in March, April and May were considered as thermo-neutral conditions.

### Growth performance

The BW was higher (p<0.001) during P4 than P1, reflecting animal age. Moreover, the daily concentrate intake was higher (p = 0.03) during P4 than P1 (Table 3). In this study, the daily allowance of concentrate was set at 1.6% BW and was adjusted each month based on BW. Thus, the higher concentrate intake during P4 reflected the increased concentrate allowance, corresponding to a higher BW during P4 than P1. Daily forage intake was higher in P1 than in the other months. RPF supplementation did not affect concentrate and roughage intake during all studied months. Neither month nor RPF supplementation affected the average daily gain and feed efficiency (gain-to-feed ratio; Table 3). In a previous study, dietary RPF supplementation (4.5% fatty acid calcium salts) increased the G:F ratio in beef cattle [13]. In South Korea, commercial feed for Korean cattle steers has generally higher energy levels to produce highly marbled beef [21]. Additional energy supply through RPF supplementation may not have significant impact on growth performance in mild cold conditions. Another possible explanation for no influence on growth performance by fat addition is an insufficient amount of RPF supplementation (0.5%).

### Rumen volatile fatty acids and NH_3_-N

Neither month nor RPF supplementation affected ruminal pH (p>0.05; Table 4). However, ruminal NH_3_-N concentrations were higher (p<0.05) in cold winter (February) than spring (April and May) (Table 4). For comparison, in sheep, NH_3_ production was dependent on diet and ambient temperature [22]. Moreover, cold exposure did not affect ruminal NH_3_ production with barley–canola seed meal and lucerne diets, but reduced NH_3_ production with a bromegrass diet. Meanwhile, cold exposure reduced the irreversible loss of both plasma urea and rumen NH_3_, and the conversion of plasma urea-nitrogen into rumen NH_3_ was greater in cold-exposed sheep than in warm sheep [23]. The same authors reported an increased efficiency of ruminal microbial synthesis under cold exposure in sheep. Thus, the differences in rumen NH_3_ concentrations between cold winter and spring in this study may have been related to changes in the irreversible loss of NH_3_, conversion of urea into NH_3_, or to the efficiency of microbial synthesis upon cold exposure. Meanwhile, RPF supplementation did not affect ruminal NH_3_-N concentrations.

Neither month nor RPF supplementation affected C2, C3, iso-C5, C5, or total VFA concentrations in rumen fluid (p> 0.05; Table 4). The C2:C3 ratio tended to be lower (p = 0.07) in the RPF supplementation group than in the control group. In another study, increasing levels of protected lipids linearly increased the molar proportion of C3, whereas the molar proportion of C2 remained unchanged, resulting in a linear decrease in the C2:C3 ratio [24]. RPF supplementation decreased (p = 0.02) ruminal iso-C4 concentrations, while ruminal C4 concentrations varied by season (p = 0.02; Table 4).

### Blood cortisol and metabolites

Plasma cortisol concentrations were lower (p<0.05) on the starting day than during the other periods, and RPF supplementation did not affect (p>0.05) cortisol concentrations (Table 5). For comparison, plasma cortisol and corticosterone concentrations were similar among newborn calves at −4.0°C and 16°C, although the concentrations varied at −12°C and −18°C in one or two animals [25]. Collectively, blood cortisol concentration does not appear to be a suitable marker of cold stress, although cortisol is commonly used as a general stress marker [26].

Serum glucose concentrations were generally higher (p< 0.001) in colder weather but were unaffected by RPF supplementation (Table 5). Young [27] suggested that increased blood glucose results from increased metabolism (e.g., metabolic rate or heart rate) under cold conditions. In humans, lipid and muscle glycogen have major roles in providing the energy required for heat production under cold exposure, whereas plasma glucose has only a minor role [28]. Meanwhile, in lactating beef cows, fat supplementation did not affect circulating glucose concentrations [29].

Month did not affect total cholesterol concentrations (p> 0.05; Table 5), and serum HDL concentrations were similar among January, February, March, and early April, albeit that the concentrations in late April were comparatively lower. RPF supplementation increased both total cholesterol (p = 0.004) and HDL concentrations (p = 0.03; Table 5). In Holstein calves, various types of fat supplementation increased plasma cholesterol and HDL concentrations, with no differences seen in TG and glucose concentrations, during the cold season [30]. Furthermore, dietary rumen-protected oleic acid increased blood HDL concentrations in cattle [31]. Collectively, the increased HDL concentrations observed in this and previous studies may be due to increased amounts of absorbed fat in the small intestine via RPF supplementation. Moreover, changes in posthepatic lipoprotein metabolism, including alterations in HDL concentrations, may contribute to increased cholesterol concentrations with RPF supplementation, as suggested by Duske et al [32].

Neither month nor RPF supplementation affected serum TG, LDL, or NEFA concentrations. The effects of RPF supplementation on blood NEFA concentrations in several studies of lactating dairy cows were inconsistent with our results [24,33], and showed an increase in blood NEFA concentrations with RPF supplementation. Overall, little information is available on the effects of RPF supplementation on NEFA concentrations in beef cattle.

Serum albumin concentrations were higher (p = 0.017) on the experimental starting day in the colder month (January) than in the other months in spring, although the concentrations were unaffected by RPF supplementation (p>0.05; Table 4). A previous study revealed higher serum albumin concentrations with changes in the osmotic pressure of body fluid during hotter summer periods than colder winter periods in dairy cows [33]. Thus, the seasonal variation in circulating albumin concentrations remains unclear.

Serum GOT and GPT concentrations were not affected by month, though GPT concentrations were lower (p<0.001) in the RPF supplementation group, which has not been reported previously (Table 5).

Month and/or RPF supplementation affected blood mineral concentrations (Table 5). Blood calcium concentrations were higher (p<0.001), while blood magnesium concentrations were lower (p<0.001), on the starting day in January compared with the spring months. In addition, the RPF supplementation group had lower (p<0.001) phosphorus and magnesium concentrations than the control group. In a previous study, serum magnesium levels were lower in hyperthyroid human patients [34]. Furthermore, thyroid hormone thyroxine increased under cold conditions in beef cattle [35]. Thus, the lower magnesium concentrations under cold conditions in this study may have been indirectly influenced by higher circulating thyroid hormone levels. However, changes in these mineral concentrations according to RPF supplementation have not been well studied.

## CONCLUSION

Growth performance (i.e., average daily gain and gain-to-feed ratio) remained unchanged during all studied months (January to May). Furthermore, rumen fermentation parameters, such as C2, C3, and total VFA concentrations, were unaffected by cold conditions and RPF supplementation, although NH_3_-N concentrations were altered under cold conditions. Serum glucose and HDL concentrations were generally higher under cold conditions; however, other serum lipid metabolites (e.g., TG, LDL, and NEFA) did not differ among the studied months. These results suggest that Korean cattle may not have been significantly affected by cold conditions in this study, although cold conditions partially affected rumen fermentation and blood parameters. Moreover, RPF supplementation did not affect growth performance or major rumen VFA (C2, C3, and C4) concentrations, although RPF supplementation resulted in altered cholesterol and HDL concentrations. Further research is warranted to clarify which cold conditions significantly affect the growth performance of Korean cattle.

## Figures and Tables

**Table 1:** Ingredients of the concentrate and composition of experimental diets for Korean cattle steers

Items	Control concentrate	RPF concentrate		Control concentrate	RPF concentrate
	
Ingredients (% DM)			Chemical composition of concentrates (% DM)		
Ground corn	1.48	0.68	DM	88.46	88.25
Wheat bran	25.00	25.00	Moisture	11.54	11.75
Rice bran	4.88	5.00	CP	14.50	14.50
Soy hull	8.00	8.00	EE	3.63	5.30
Urea	0.39	0.35	Ash	9.98	7.93
Salt	0.20	0.20	NDF	27.26	27.60
Molasses	3.50	3.50	ADF	11.54	11.75
Whole cottonseed	3.00	3.00	NFC	34.28	34.40
Cottonseed hull	3.00	3.00	Ca	1.45	1.32
Bentonite	0.30	0.30	P	0.45	0.46
Magnesium oxide	0.30	0.30	Crude fiber	8.93	9.23
Sodium bicarbonate	0.45	0.45	ME3) (Mcal/kg)	2.47	2.63
Steamed–flaked corn	21.00	21.00	NE4) (Mcal/kg)	1.12	1.22
Corn flour	5.48	7.61	Chemical composition of rice straw (% DM)		
Condensed molasses soluble	1.20	1.20	DM	74.91	
Coconut meal	5.00	5.00	CP	3.87	
Rapeseed meal	2.41	2.04	EE	1.37	
Wheat flour	5.00	5.00	Ash	11.46	
Distiller’s dried grains with solubles	0.00	0.61	Ca	0.25	
Corn gluten feed	4.78	3.88	P	0.095	
Limestone	2.89	2.57	ADF	51.97	
Live yeast	0.07	0.01	NDF	31.52	
Barley stone	1.18	0.00			
Rumen protected fat	0.00	0.50			
Palm oil	0.30	0.61			
Mineral/vitamin premix1)	0.19	0.19			
Total	100.00	100.00			
TDN2)	71.79	75.36			

DM, dry matter; RPF, rumen-protected fat; TDN, total digestible nutrients; CP, crude protein; EE, ether extract; NDF, neutral detergent fiber; ADF, acid detergent fiber; NFC, non-fiber carbohydrate; ME, metabolizable energy; NE, net energy.

1) Mineral and vitamin premix contained vit A 2,650,000 IU, vit D_3_ 530,000 IU, vit E 1,050 IU, niacin 10,000 mg, Mn 4,400 mg, Zn 4,400 mg, Fe 13,200 mg, Cu 2,200 mg, iodine 440 mg, and Co 440 mg/kg of Grobic–DC. Grobic–DC was provided by Bayer Health Care (Leverkusen, Germany).

2) TDN (%) = NFC+CP+[(EE–1)×2.25]+NDF–7 [10].

3) ME = [1.01×(DE)–0.45]+0.0046×(EE–3) [10].

4) NE = 1.42 ME–0.174 ME^2^+0.0122 ME^3^–1.65 [10].

**Table 2:** Mean, maximum, and minimum values of ambient temperatures, climate temperatures, and relative humidity during January through April of 2015

Items	January (P1)1)	February (P2)1)	March (P3)1)	April (P4)1)	SE	p-value
Ambient temperature (°C)						
Mean	−3.44^a^	−0.70^b^	5.87^c^	11.18^d^	0.58	<0.001
Maximum	4.49^a^	5.04^a^	17.78^b^	20.68^c^	1.53	<0.001
Minimum	−9.40^a^	−5.5^b^	−1.86^c^	4.28^d^	0.49	<0.001
Climate temperature (°C)						
Mean	−2.91^a^	−0.24^b^	4.68^c^	10.36^d^	0.58	<0.001
Maximum	8.78^b^	7.00^a^	16.16^c^	18.12^d^	1.59	<0.001
Minimum	−10.57^a^	−5.78^b^	−3.12^c^	3.88^d^	0.54	<0.001
Ambient relative humidity (%)						
Mean	75.32	66.35	52.33	57.64	2.12	0.31
Maximum	90.03	83.38	89.95	77.90	2.60	0.27
Minimum	50.51^b^	45.92^b^	19.03^a^	35.95^ab^	2.69	0.012
Climate relative humidity (%)						
Mean	71.39	62.26	54.34	75.37	2.44	0.08
Maximum	91.80	81.42	93.55	106.38	3.36	0.51
Minimum	40.08^b^	38.80^b^	18.39^a^	44.36^b^	3.17	<0.001

n = 28.

SE, standard error.

1) P1, January 9 to February 5 (4 weeks); P2, February 6 to March 5 (4 weeks); P3, March 6 to April 3 (4 weeks); P4, April 4 to May 2 (4 weeks).

a–d Mean values with different letters differ at p<0.05.

**Table 3:** Growth performance of Korean cattle steers fed either control or RPF–supplemented diet during January through April of 2015

Items	January (P1)1)		February (P2)1)		March (P3)1)		April (P4)1)		SE	p-value		
				
	Control	RPF	Control	RPF	Control	RPF	Control	RPF		Month	Diet	Interaction
Initial body weight (kg)	547.2	554.0	571.6	579.0	587.2	595.6	604.4	616.0	5.28	<0.001	0.34	0.87
Body weight (kg)	571.6	579.0	587.2	595.6	604.4	616.0	625.2	636.8	5.57	<0.001	0.35	0.94
Daily feed intake (kg: DM base)												
Total feed intake (kg/d)	7.52	7.43	7.24	7.38	7.59	7.51	7.66	7.76	0.07	0.12	0.92	0.75
Concentrate intake (kg/d)	6.66	6.57	6.50	6.64	6.84	6.76	6.91	7.01	0.07	0.03	0.93	0.74
Forage intake (kg/d)	0.86	0.86	0.74	0.74	0.75	0.75	0.75	0.75	0.01	<0.001	0.98	0.99
Average daily gain (kg/d)	0.87	0.89	0.56	0.59	0.61	0.73	0.77	0.77	0.03	0.39	0.41	0.62
Feed efficiency (G:F ratio)	0.104	0.124	0.077	0.080	0.080	0.097	0.099	0.098	0.01	0.35	0.33	0.60

n = 10/group.

RPF, rumen-protected fat; SE, standard error.

1) P1, January 9 to February 5 (4 weeks): a mild cold stress range; P2, February 6 to March 5 (4 weeks): a mild cold stress range; P3, March 6 to April 3 (4 weeks): a thermo-neutral range; P4, April 4 to May 2 (4 weeks): a thermo-neutral range.

**Table 4:** Ruminal pH, VFAs and NH_3_-N of Korean cattle steers fed either control or RPF–supplemented diet during January through April of 2015

Items	February 6		March 5		April 3		May 2		SE	p-value		
				
	Control	RPF	Control	RPF	Control	RPF	Control	RPF		Month	Diet	Interaction
Temperature1) (°C)	−0.26		6.79		10.8		14.0					
pH	7.21	7.04	6.74	6.50	6.73	6.77	6.85	6.90	0.06	0.46	0.50	0.17
NH_3_-N (mg/dL)	13.30	8.97	11.06	13.59	5.19	4.57	7.42	3.46	0.69	<0.001	0.40	0.45
C2 (mM)	36.22	37.84	51.24	52.71	43.89	46.06	46.19	39.34	1.12	0.82	0.87	0.18
C3 (mM)	10.25	10.98	15.81	17.39	14.48	18.13	13.60	12.63	0.54	0.47	0.22	0.59
C2:C3 ratio	3.62	3.46	3.29	3.11	3.14	2.84	3.46	3.14	0.07	0.24	0.07	0.66
iso-C4 (mM)	0.71	0.65	0.88	0.81	0.74	0.71	0.86	0.68	0.02	0.63	0.02	0.22
C4 (mM)	10.28	10.01	13.99	14.99	9.79	10.66	10.27	8.38	0.40	0.02	0.98	0.34
iso-C5 (mM)	1.10	1.15	1.51	1.27	1.19	1.06	1.36	0.98	0.05	0.62	0.08	0.27
C5 (mM)	0.79	0.80	1.33	1.37	0.98	1.18	1.10	0.91	0.04	0.87	0.76	0.40
Total VFA (mM)	59.35	61.43	84.76	88.55	71.07	77.81	73.38	62.93	1.86	0.86	0.84	0.22

n = 10/group. February 6 is within a mild cold stress range, and other 3 days are within a thermo-neutral range.

VFA, volatile fatty acid; RPF, rumen-protected fat; SE, standard error.

1) Average ambient temperatures of sampling times of each day.

**Table 5:** Plasma cortisol and serum parameters of Korean cattle steers fed either control or RPF–supplemented diet during January through April of 2015

Items	January 9		February 6		March 5		April 3		May 2		SE	p-value		
					
	Control	RPF	Control	RPF	Control	RPF	Control	RPF	Control	RPF		Month	Diet	Interaction
Temperature1) (°C)	−6.42		−0.26		6.79		10.8		14.0					
Cortisol (μg/dL)	35.6	37.6	64.7	62.2	57.0	41.2	54.3	54.0	59.0	64.5	5.68	0.012	0.55	0.65
Glucose (mg/dL)	74.3	72.7	78.9	75.5	76.1	75.6	66.7	66.9	59.0	57.8	0.90	<0.001	0.34	0.75
Cholesterol (mg/dL)	170.1	174.6	147.4	168.3	156.1	168.5	154.7	178.4	152.1	180.9	3.09	0.78	0.004	0.21
Triglyceride (mg/dL)	14.8	13.6	21.0	20.0	14.4	15.7	16.0	15.8	15.8	15.2	0.43	0.70	0.84	0.76
HDL (mg/dL)	101.7	103.9	97.7	105.3	94.3	102.3	93.8	102.1	87.8	92.5	1.39	0.003	0.03	0.75
LDL (mg/dL)	23.7	21.9	17.3	18.7	19.1	19.3	19.3	21.7	21.4	25.4	0.61	0.31	0.51	0.13
NEFA (mg/dL)	303.8	283.0	193.0	271.4	439.2	374.1	296.4	308.9	264.6	280.7	11.39	0.95	0.97	0.88
Albumin (mg/dL)	3.51	3.56	3.36	3.47	3.39	3.53	3.38	3.39	3.38	3.38	0.02	0.017	0.18	0.52
GOT (IU/dL)	73.5	71.0	75.9	79.7	79.2	78.2	74.2	72.3	74.2	74.8	1.16	0.86	0.99	0.92
GPT (IU/dL)	26.1	23.6	24.1	22.5	25.1	22.2	24.1	21.1	24.6	22.7	0.35	0.18	<0.001	0.95
Calcium (mg/dL)	9.69	9.77	9.47	9.40	9.37	9.78	9.30	9.31	9.34	9.25	0.04	<0.001	0.40	0.58
Phosphorus (mg/dL)	7.92	7.68	8.70	7.95	8.09	7.62	7.83	7.19	8.60	7.84	0.08	0.75	<0.001	0.40
Magnesium (mg/dL)	2.56	2.39	2.74	2.48	2.82	2.63	2.83	2.44	2.89	2.65	0.03	<0.001	<0.001	0.40

n = 10/group. January 9 and February 6 are within a mild cold stress range, and other 3 days are within a thermos-neutral range.

RPF, rumen-protected fat; SE, standard error; HDL, high density lipoprotein; LDL, low density lipoprotein; NEFA, non-esterified fatty acid; GOT, glutamic oxalacetic transaminase; GPT, glutamic pyruvate transaminase.

1) Average ambient temperatures of sampling times of each day.
